# Impaired myocardial perfusion on CMR is associated with increased numbers of classical monocytes in STEMI patients treated by primary PCI

**DOI:** 10.1186/1532-429X-13-S1-O93

**Published:** 2011-02-02

**Authors:** Lourens FHJ Robbers, Anja M van der Laan, Robin Nijveldt, Aernout M Beek, Ronak Delewi, Alexander Hirsch, C Ellen van der Schoot, Bart J Biemond, Felix Zijlstra, Jan J Piek, Albert C van Rossum

**Affiliations:** 1 VU University Medical Center, Amsterdam, Netherlands; 2 Academic Medical Center, Amsterdam, Netherlands; 3 Sanquin Research and Landsteiner Laboratory, Amsterdam, Netherlands; 4 University Medical Center Groningen, Groningen, Netherlands

## Introduction

Despite optimal revascularization strategies, STEMI patients often show impaired myocardial tissue reperfusion due to microvascular obstruction. Monocytes, especially the classical monocytes have been suggested to contribute to microvascular obstruction through a complex inflammatory response.

## Purpose

To investigate the relation between the circulating number of classical monocytes and severity of myocardial perfusion impairment in patients with ST-elevated myocardial infarction (STEMI) treated with primary PCI.

## Methods

Fifty-four patients underwent CMR first-pass perfusion imaging, late gadolinium enhancement (LGE) and functional imaging between 3-7 days after primary PCI.

Myocardial perfusion in the infarct core was evaluated semi-quantitatively from signal intensity versus time curves by calculation of the maximum upslope relative to a remote, normal segment. The number of classical (CD14^++^CD62L^+^) (CM) and nonclassical (CD14^+^CD62L^-^) monocytes (NCM) was measured by flow cytometry from a venous blood sample. Patients were divided in tertiles according to the monocyte count in the sample.

## Results

**Figure 1:**
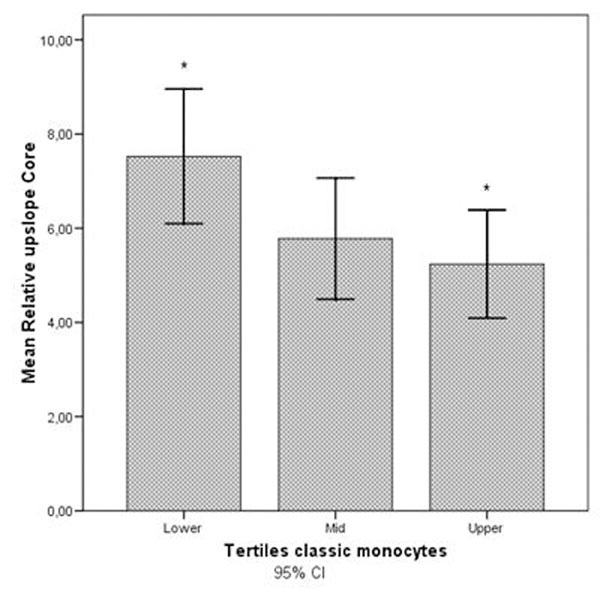
* Significant difference in relative upslope value between lower and upper tertiles (p=0.014).

Myocardial perfusion in the infarct core was significantly lower in patients with high numbers of CM (upper tertile) compared to low numbers (lower tertile) of CM (mean relative upslope in upper tertile 7.5 versus mid tertile 5.8 versus lower tertile 5.2, p=0.014). Also, patients with high numbers of CM had a larger infarct size (9.17 vs. 6.51 vs. 3.89 g/m^2^, p<0.001), more segments with microvascular obstruction at LGE (15.9 vs. 8.2 vs. 2.2% of segments, p=0.001), and greater impairment of ejection fraction (37.0 vs. 42.0 vs. 46.5%, p=0.001) than patients with low numbers of CM. No such relations were found for NCM. Figure 1.

## Conclusions

In reperfused STEMI patients, high numbers of classical monocytes are associated with impaired myocardial perfusion, increased myocardial injury and greater functional loss. This suggests a causal relationship between circulating monocytes after AMI and degree of myocardial damage in reperfused infarction.

